# The revised Addenbrooke's Cognitive Examination can facilitate differentiation of dementia with Lewy bodies from Alzheimer's disease

**DOI:** 10.1002/gps.5483

**Published:** 2020-12-14

**Authors:** Maria Angeles Prats‐Sedano, George Savulich, Ajenthan Surendranathan, Paul C. Donaghy, Alan J. Thomas, James B. Rowe, Li Su, John T. O'Brien

**Affiliations:** ^1^ Department of Psychiatry School of Clinical Medicine University of Cambridge Cambridge UK; ^2^ Translational and Clinical Research Institute Newcastle University Newcastle upon Tyne UK; ^3^ Department of Clinical Neurosciences School of Clinical Medicine University of Cambridge Cambridge UK

**Keywords:** Addenbrooke's Cognitive Examination‐revised, Alzheimer's disease, dementia with Lewy bodies, diagnosis, neurodegeneration, neuropsychology

## Abstract

**Objectives:**

Dementia with Lewy bodies (DLB) is a major cause of degenerative dementia, yet the diagnosis is often missed or mistaken for Alzheimer's disease (AD). We assessed whether the revised Addenbrooke's Cognitive Examination (ACE‐R), a brief test for dementia, differentiates DLB from AD.

**Methods:**

We first compared baseline ACE‐R performance in 76 individuals with DLB, 40 individuals with AD and 66 healthy controls. We then investigated the diagnostic accuracy of a simple standardised ‘memory/visuospatial’ ratio calculated from the ACE‐R subscores. Finally, as a comparison a logistic regression machine learning algorithm was trained to classify between DLB and AD.

**Results:**

Individuals with AD had poorer memory (*p* = 0.001) and individuals with DLB had poorer visuospatial function (*p* = 0.005). Receiver operating characteristics curves confirmed that the ACE‐R total score could differentiate dementia from non‐dementia cases with 98% accuracy, but could not discriminate between dementia types (50%, or chance‐level accuracy). However, a ‘memory/visuospatial’ ratio ≥1.1 differentiated DLB from AD with 82% sensitivity, 68% specificity and 77% mean accuracy. The machine learning classifier did not improve the overall diagnostic accuracy (74%) of the simple ACE‐R subscores ratio.

**Conclusions:**

The ACE‐R‐based ‘memory/visuospatial’ ratio, but not total score, demonstrates good clinical utility for the differential diagnosis of DLB from AD.

## INTRODUCTION

Dementia with Lewy bodies (DLB) is characterised by recurrent visual hallucinations, parkinsonian motor symptoms, rapid eye movement (REM) sleep behaviour disorder and fluctuating cognitive impairment.^1^ Although DLB is one of the major causes of degenerative dementia, early diagnosis remains challenging. Alzheimer's disease (AD) and movement disorders are among the most frequent misdiagnoses, largely due to shared symptomology (e.g., spontaneous extrapyramidal motor features^2^) and difficulty to detect cognitive impairment early. Identifying the presence of DLB by clinical assessment is further complicated by dementia due to co‐occurring AD.^3^ Overlapping neuropsychiatric symptoms can also lead to misdiagnosis.^4^ Individuals with DLB may benefit from the treatment of Parkinsonism or other autonomic symptoms, but adversely react to neuroleptics, with increased morbidity and mortality in severe cases.^5^ DLB specific management pathways have recently been developed,^6^ but an accurate diagnosis is clearly needed for individuals to benefit from these.

**Key points**

- Discriminating between dementia with Lewy bodies (DLB) and Alzheimer's disease (AD) is challenging, especially in early stages
- A ‘memory/visuospatial’ ratio calculated from the widely used revised Addenbrooke's Cognitive Examination (ACE‐R) accurately differentiated DLB from AD, whereas the ACE‐R total score performed at chance level
- Routinely collected cognitive assessment provides a brief and easily accessible method for assisting clinical diagnosis
- Improving diagnostic accuracy has several advantages for individuals with DLB and their carers, including better provision of support services and disease specific management

Neuropathological examination has indicated that around 50% of cases with DLB pathology presented with global impairments typical of AD, leading to considerable under‐diagnosis.^2^ Individuals with DLB are given more prior alternative diagnosis, undertake more brain scans and experience longer delays before receiving a final diagnosis than other dementia types.^7^ Cognitive assessment provides a reliable and domain‐specific profile of impairment. Memory decline associated with medial temporal atrophy is highly characteristic of AD, whereas deficits in visuospatial function compared to relatively intact memory and object naming are more pronounced in DLB.^1^, ^8^ Given the complex clinical heterogeneity of DLB and considerable variation in regional diagnostic rates,^9^ comprehensive examination is required for making a probable diagnosis (i.e., the presence of dementia with at least two core features^1^). The potential utility of cognitive markers for minimising the number of false*‐*negative and false‐positive cases is less clear. In a cohort with established accuracy of a clinical diagnosis verified against post‐mortem evaluation, a memory to praxis ratio derived from subscales of the cognition section (CAMCOG^10^) of the Cambridge Examination for Mental Disorders of the Elderly showed 63% sensitivity and 84% specificity at the optimal cut‐off score (≥0.4) for discriminating DLB from AD.^11^ However, the value of other brief and widely used cognitive scales has not been reported.

The revised Addenbrooke's Cognitive Examination^12^ (ACE‐R) is a brief cognitive screening assessment that is sensitive to the early stages of dementia and able to differentiate between dementia subtypes.^12^, ^13^ Modifications to the original version were made to facilitate easier administration, remove insensitive items and include parallel versions of the name and address recall. The Addenbrooke's Cognitive Examination‐Revised (ACE‐R) also includes more tests of visuospatial abilities relevant to cognitive impairment in DLB than the CAMCOG and Mini‐Mental State Examination (MMSE).^14^ Increased sensitivity and specificity of the ACE‐R has been partly attributed to expansion of the visuospatial domain.^12^ This is particularly important, as others have suggested that clinical interpretation of the ACE‐R should be guided by its latent‐variable structure, in which visuospatial abilities have been identified as a notable factor.^15^ Previous studies have used the ACE‐derived ‘Verbal + Language/Orientation + Memory (VLOM)’ ratio to discriminate frontotemporal dementia (FTD) from AD, with mixed success.^13^, ^16^, ^17^ Others have used the verbal fluency subscore for the differential diagnosis of parkinsonian syndromes^18^ and the total ACE‐R score to differentiate between AD and late‐life depression.^19^ Yet no studies to date have used this instrument to distinguish DLB from other dementia types. To redress this, we calculated a simple standardised ‘memory/visuospatial’ ratio similar to Ballard and colleagues,^11^ but using the ACE‐R subscores most likely to discriminate between DLB and AD.

Another approach for combining subscores would be to apply machining (ML) techniques. As cognition is multivariate, the traditional reliance on univariate tests weakens the ability to detect group differences. For example, subscores in combination may be much more sensitive than when considered in isolation. ML has been previously used to ‘train’ models that can detect and differentiate between pathologies.^20^, ^21^ An established ML algorithm used for classification is logistic regression.^22^ Using linear rather than deep learning models allows for features underpinning any group discrimination to be readily understood. This affords a good balance of inference power (accuracy) and transparency (interpretability). For this reason we considered logistic regression as a good model comparator. Performance of our ‘memory/visuospatial’ ratio could thus be interpreted against commonly used predictive modelling. Assuming equal performance of the two models, the advantage of our ratio is that it is much easier to use than a ML classifier (e.g., quicker to calculate; can be calculated with paper and pencil using raw scores; no specialist training is required).

We first aimed to replicate the clinical utility of the ACE‐R for differentiating between dementia and non‐dementia in our sample. Secondly, using receiver operating characteristics (ROCs) curves, we tested the hypothesis that the ‘memory/visuospatial’ ratio would discriminate DLB from AD. As a comparison, we used a ML classifier with the ACE‐R subscales as features and compared its overall mean accuracy with the overall mean accuracy of the ‘memory/visuospatial’ ratio. Establishing the accuracy of the ‘memory/visuospatial’ ratio for the differential diagnosis of DLB from AD could lead to its use in memory clinic and dementia assessment settings, whereby a simple and easily calculated score could assist with clinical diagnosis, inform the most appropriate treatment strategy and optimise disease‐specific support services.

## MATERIALS AND METHODS

### Participants

Participant data were obtained from baseline visits of three UK dementia studies: Neuroimaging of Inflammation in Memory and Other Disorders^23^ (NIMROD, 13/EE/0104, Cambridge); the Multimodal Imaging of Lewy Body Disorders study (MILOS, 16/EE/0531, Cambridge); and Amyloid Imaging for Phenotyping Lewy Body Dementia^24^ (AMPLE, 13/NE/0064, Newcastle). Volunteers with Lewy body dementia met either the 2005 consensus criteria^25^ (NIMROD, AMPLE) or the 2017 revised criteria^26^ (MILOS) for probable DLB. Participants with AD met the diagnostic criteria for probable AD as defined by the National Institute of Neurological and Communicative Disorders and Stroke (NINCDS) and the AD and Related Disorders Association (ADRDA).^27^ Control participants were healthy adults with an absence of regular memory problems, signs or symptoms suggestive of dementia (including MMSE^28^ score >26) or significant medical illnesses. Participants were aged 50 years and older. Exclusion criteria were any co‐existing neurological conditions and a history of substance dependence. All participants provided informed written consent.

### Addenbrooke's Cognitive Examination‐Revised

The ACE‐R^12^ is an objective and reliable 100‐point test that evaluates multiple cognitive domains: orientation/attention (18‐points), memory (26‐points), verbal fluency (14‐points), language (26‐points) and visuospatial ability (16‐points). The memory subscale comprises items of semantic and episodic content (e.g., recall, anterograde, recognition) and the visuospatial subscale includes copying overlapping pentagons, copying a wire cube and free drawing a clock. The ACE‐R has shown good reliability (Cronbach's alpha = 0.08) with two‐cut off scores previously identified for detecting people with dementia (88/100: sensitivity = 0.94, specificity = 0.89 and 82/100: sensitivity = 0.84, specificity = 1.0); the likelihood of having dementia at the latter score was 100:1^12^. The test takes approximately 20 min to complete.

### Statistical analyses and predictive modelling

Basic demographic information was analysed using one‐way analysis of variance, independent samples t‐tests and chi‐square tests as appropriate. Due to non‐normal distributions and highly skewed cognitive data, the ACE‐R subscales and total score were *Z‐*transformed and analysed using Kruskal–Wallis H tests (for three‐group comparisons) and Mann–Whitney U tests (clinical group comparisons only). Data were analysed using SPSS version 25.

A logistic regression ML model^29^ was trained to classify between DLB and AD, excluding the healthy control participants. The model was written as follows:

$$log\;odds\;\text{DLB}\;\left(\text{scores}\right)\;=\;w_{attention}*s_{attention}+\;w_{memory}*s_{memory}+w_{verbal\;fluency}*\;s_{verbal\;fluency}+\;w_{language}*s_{language}+\;w_{visuospatial}*\;s_{visuospatial}$$

where, each *s* is an ACE‐R subscore (orientation/attention, memory, verbal fluency, language and visuospatial ability) acting as a feature in the model and each *w* is its associated weight. Disease probability can then be computed by applying a sofmax function:

$$P\left(\text{DLB}\right)=\frac{e^{\log\;odds\;\text{DLB}}}{1+e^{\log\;odds\;\text{DLB}}},\;P\left(\text{AD}\right)=1-P\left(\text{DLB}\right)$$

where, $e^{x}$ is the natural exponential function. The final prediction is then obtained by setting a threshold (i.e., when the probability of DLB is higher than 0.5, the model predicts DLB, and vice versa for AD). Each subscore is normalised by subtracting the mean and dividing by the standard deviation, both obtained from the full dataset. In order to report diagnostic accuracy that generalises well, we used a L2 regulariser with a coefficient *C* = 1 on each weight during training, and trained the model using 85% of the data (randomly selected). We then evaluated the accuracy, sensitivity and specificity of the classifier using the remaining 15% of the data (test set). This process was repeated with 20 different random seeds. All procedures were coded in Python version 3 and tested using the machine learning toolbox scikit‐learn version 0.22.2.post1.

## RESULTS

### Participant characteristics

Demographic information for the DLB (*n* = 76), AD (*n* = 40) and healthy control (*n* = 66) groups are presented in Table 1. Participants were predominantly male (73.6%) with a mean age of 73.8 years (SD = 7.10). The three‐groups were well matched for age, but sex ratio and years in education were significantly different. However, sex ratio and years in education did not significantly differ between the DLB and AD groups. Participants in these groups were in mild‐to‐moderate disease stages, as reflected by total ACE‐R and MMSE scores.

**TABLE 1:** Demographic characteristics and ACE‐R performance (means and standard deviations) by group

	DLB *n* = 76	AD *n* = 40	HC *n* = 66	3‐group comparisons	DLB versus AD
Demographics					
Age (years)	74.8 (6.3)	73.8 (8.6)	72.6 (6.9)	*F* (2,179) = 1.88, *p* = 0.16	*t* (61.90) = 0.69, *p* = 0.50
Education (years)	11.8 (3.1)	12.5 (2.9)	14.1 (3.4)	*F* (2,179) = 9.80, *p* < 0.001	*t* (114) = −1.15, *p* = 0.25
Sex (male: female)	64: 12	28: 12	42: 24	*X* ^*2*^ = 8.05, *p* = 0.02	*X* ^*2*^ = 2.97, *p* = 0.09
MMSE	22.7 (4.4)	22.3 (1.13)	28.9 (1.1)	***X*** ^***2***^ **(2) = 104.53 *p* < 0.001**	*U* = 1467.00, *p* = 0.76
ACE‐R					
Attention/orientation	14.1 (3.3)	14.3 (3.6)	17.9 (0.4)	***X*** ^***2***^ **(2) = 83.77, *p* < 0.001**	*U* = 1431.00, *p* = 0.60
Memory	14.9 (5.2)	11.5 (5.3)	23.6 (2.5)	***X*** ^***2***^ **(2) = 106.60, *p* < 0.001**	***U* = 973.50, *p* = 0.001**
Fluency	6.2 (3.1)	6.6 (3.2)	11.7 (2.1)	***X*** ^***2***^ **(2) = 89.36, *p* < 0.001**	*U* = 1390.50, *p* = 0.45
Language	22.3 (2.9)	22.1 (3.8)	25.1 (1.0)	***X*** ^***2***^ **(2) = 51.17, *p* < 0.001**	*U* = 1469.50, *p* = 0.77
Visuospatial	10.2 (3.9)	12.3 (3.4)	15.6 (0.8)	***X*** ^***2***^ **(2) = 91.13, *p* < 0.001**	***U* = 1041.50, *p* = 0.005**
Total	67.9 (14.2)	66.7 (15.5)	93.9 (4.7)	***X*** ^***2***^ **(2) = 115.25, *p* < 0.001**	*U* = 1519.50, *p* = 0.10

*Note*: Entries in bold indicates significant findings.

Abbreviations: ACE‐R, revised Addenbrooke’s Cognitive Examination; AD, Alzheimer’s disease; DLB, Dementia with Lewy bodies; HC, Healthy controls; MMSE, Mini‐Mental State Examination.

### Differentiating dementia and non‐dementia participants

**FIGURE 1:**
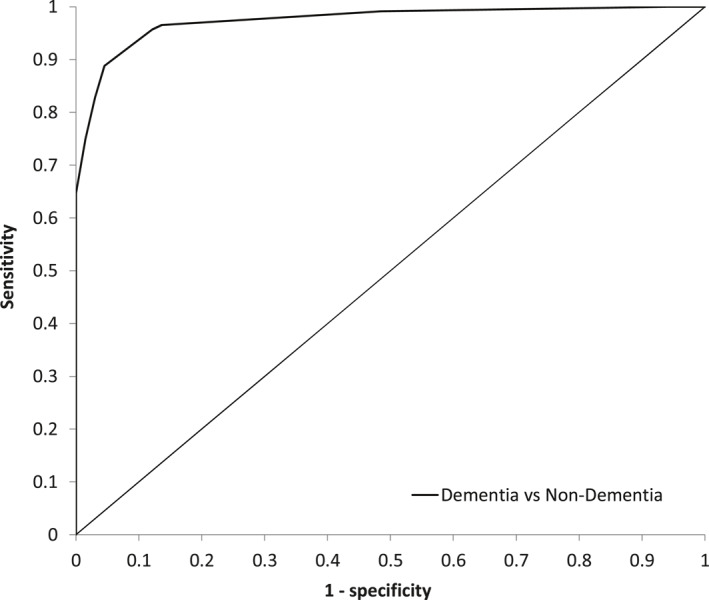
The ROC curve plotting the trade‐off between the true positive rate (sensitivity) and false positive rate (1—specificity) showed strong clinical utility of the ACE‐R total score for detecting dementia (AUC = 0.98); Abbreviations: ACE‐R, revised Addenbrooke's Cognitive Examination; AUC, area under the curve; ROC, receiver operating characteristics

One hundred sixteen individuals with dementia and 66 control participants were included in the model. An ACE‐R cut‐off score of 88/100 showed optimal sensitivity (96%) and specificity (88%) for identifying dementia from non‐dementia. Similarly, a cut‐off score of 82/100 revealed high sensitivity (83%) and greater specificity (97%.) The ROC curve plotting the trade‐off between the true positive rate (sensitivity) and false positive rate (1—specificity) showed strong clinical utility of the ACE‐R total score for detecting dementia (area under the curve, AUC = 0.98) (Figure 1).

### Differentiating DLB from AD with the ‘memory/visuospatial’ ratio

**FIGURE 2:**
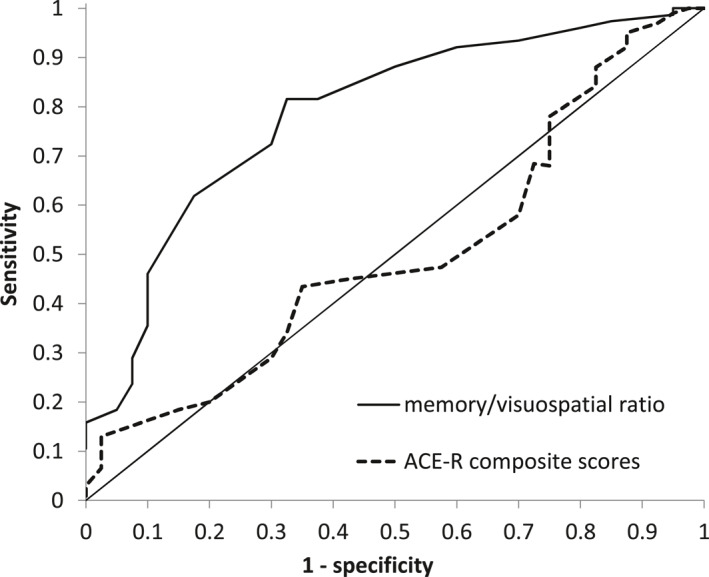
The ‘memory/visuospatial ratio showed good accuracy for detecting DLB (AUC = 0.79); the ACE‐R total score performed at chance level (AUC = 0.50). A cut‐off score of 1.1 differentiated DLB from AD with 82% sensitivity, 68% specificity, 82% positive predictive value and 65% negative predictive value; Abbreviations: ACE‐R, revised Addenbrooke's Cognitive Examination; AD, Alzheimer's disease; AUC, area under the curve; DLB, Dementia with Lewy bodies

As expected, the MMSE total score, ACE‐R subscales and ACE‐R total score were highly significantly different between the three groups (all *p*'s < 0.001; Table 1; raw scores are presented). Follow‐up comparisons between the clinical groups revealed that, as would be expected, individuals with AD had significantly poorer memory (*p* = 0.001), whereas individuals with DLB had significantly poorer visuospatial ability (*p* = 0.005). To determine the diagnostic accuracy of these subscales, in keeping with our hypothesis, we calculated a ‘memory/visuospatial’ ratio and plotted its ROC curve along with the ROC curve for the ACE‐R total score for comparison (Figure 2). The ROC curves showed that the ‘memory/visuospatial’ ratio was a good marker for detecting DLB (AUC = 0.79). In contrast, the ACE‐R total score showed no diagnostic ability for predicting dementia subtypes (AUC = 0.50). In our sample, a memory/visuospatial score ≥1.1 highly differentiated DLB from AD, with a sensitivity of 82%, a specificity of 68%, a positive predictive value of 82% and a negative predictive value of 65%. The cut‐off of 1.1 showed an overall mean accuracy of 77% to correctly differentiate DLB from AD.

### Differentiating DLB from AD with machine learning

**FIGURE 3:**
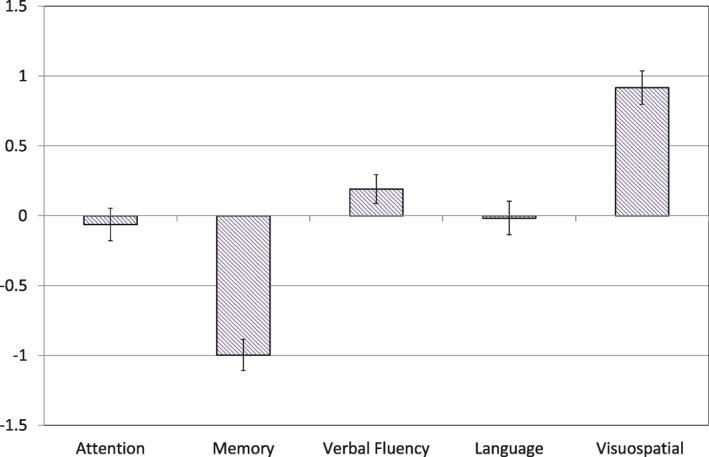
Weighted cognitive features (means and standard deviations for each ACE‐R subscore) of the logistic regression model; Abbreviation: ACE‐R, revised Addenbrooke's Cognitive Examination

We then used a logistic regression model to determine the weight of each ACE‐R subscale after training. It was shown that the memory and visuospatial subscales were the two domains that highly influenced the model, being the furthest away from zero (Figure 3). Smaller values assigned to the attention/orientation, verbal fluency and language domains indicated low influence of these features on the model. Similar to our ‘memory/visuospatial’ ratio, the logistic regression model showed 78% sensitivity, 63% specificity and 74% overall mean accuracy for differentiating between DLB and AD.

## DISCUSSION

The tendency to under‐diagnose DLB prevents appropriate treatment and disease management, which in turn increases burden on individuals with DLB and their caregivers. Higher diagnostic rates reported in secondary care than in the community likely reflects better accuracy within a specialist setting.^9^ A brief cognitive assessment such as the ACE‐R may thus provide a more easily accessible method for assisting diagnosis. We first confirmed that the ACE‐R‐total thresholds of 82 and 88 (out of 100) differentiated between dementia and non‐dementia participants with extremely high accuracy (98%). Our optimal cut‐offs were similar to those previously reported in other samples,^12^, ^13^, ^16^ replicating the validity of the ACE‐R for accurately detecting dementia. Group differences on the ACE‐R subscales revealed the expected pattern of cognitive impairment between our dementia groups, such that individuals with AD showed poorer memory and individuals with DLB showed poorer visuospatial ability. The remaining subscales did not significantly differ between the two groups. Poor memory performance in individuals with AD is largely associated with structural degeneration of the medial temporal lobe.^1^, ^8^ Early and severe deficits in visuospatial abilities have been shown to predict visual hallucinations in individuals with DLB, typically thought to reflect accumulation of alpha‐synuclein rather than AD‐related pathology.^30^ Following the memory to praxis ratio previously used to discriminate DLB from AD and vascular dementia in a consecutive cohort study,^11^ we calculated an ACE‐R derived ‘memory/visuospatial’ ratio to determine the diagnostic prediction of the observed cognitive differences. We found that the optimal cut‐off score showed good sensitivity and specificity for differentiating DLB from AD. However, the relatively low negative predictive value (i.e., number of false‐negatives) of our ‘memory/visuospatial’ ratio indicated that some individuals with AD were incorrectly classified as DLB in our sample. This may be due to a subset of individuals with AD presenting with multi‐domain cognitive dysfunction, in which lower scores assigned to impaired visuospatial ability biased classification toward DLB. As such, our ratio should be used as an extra tool alongside supporting and core clinical features (e.g., REM sleep behaviour disorder) as well as proposed biomarkers (e.g., dopaminergic abnormalities in the basal ganglia) of DLB when making a diagnosis. Importantly, the ROC curves further indicated that the total ACE‐R score had no discrimination capacity, performing only at chance level.

The ML classifier did not improve the overall diagnostic accuracy (74%) of the simple ACE‐R subscores ratio. This is likely due to the use of linear models, which generalise well and are the easiest to interpret. Although non‐linear models may give higher training performance, they are more sensitive to overfitting and suffer from lower predictive accuracy when evaluating subscores of new cases. Sensitivity and specificity of the ML classifier showed similar values to the ‘memory/visuospatial’ ratio, suggesting comparable performance between the two methods. When interpreting the weights of the ML classifier, the small values assigned to the attention/orientation, language and verbal fluency subscales support the lack of significant differences found on these subscores between our dementia groups (see Figure 3). Higher weights given to the memory and visuospatial subscores were again consistent with the expected profile of cognitive impairment and further reflect the latent‐variable structure of the ACE‐R, in which combined memory measures (anterograde retrieval and working memory items) and visuospatial ability are among the constructs best measured by the test. Including core diagnostic symptoms in our classifier, such as the presence or recent history of complex visual hallucinations, would likely have improved its discrimination threshold. However, unlike impairments in visuospatial abilities, visual hallucinations often do not manifest at initial presentation,^31^ and our aim was to assess a cognitive marker that could be calculated at any time point in the disease.

Our findings complement previous studies using the ACE‐R subscales to detect specific dementia (e.g., the ‘VLOM’ ratio for FTD^13^; the verbal fluency subscore for idiopathic PD^18^). It is worth noting that forms of standardised cognitive assessment have been used to differentiate between dementia types. For example, visual perceptual items of the Wechsler Adult Intelligence Scale‐revised and the Wechsler Memory Scale‐revised have shown to differentiate DLB from AD^32^; a ML classifier has identified poor paired associates learning as a highly accurate predictor of converting to AD^20^; and disease‐specific profiles of cognitive impairment have shown to relate to discrete signatures of gait in DLB and AD.^33^ Together, these studies indicate that objective and reliable tests of cognitive function are useful tools for illness detection and differentiation. They also provide less costly evaluation than brain imaging, and in the case of the ACE‐R, does not require specialist test equipment to administer.

There are limitations to our study. We did not separate amyloid‐positive from amyloid‐negative status, although amyloid deposition was shown not to relate to cognitive or functional impairment in a subset of our DLB sample.^24^ Inclusion of longitudinal ACE‐R data would have been useful for monitoring the rate of cognitive change (i.e., clinically significant decline) as an index of disease progression. Similarly, post‐mortem data would have allowed us to pathologically validate results from the ‘memory/visuospatial’ differentiation. As cases in our sample were mild‐to‐moderate, future work could investigate the utility of our ratio in the prodromal stages (mild cognitive impairment) of both diseases. Determining the diagnostic accuracy of the ‘memory/visuospatial’ ratio with data from the Addenbrooke's cognitive examination III (ACE‐III), which substituted items from the MMSE, would also be an important next step. We expect similar results given the same proportion of subscores and total score between the two instruments, with only one item of interest differing in the visuospatial domain (copying the intersecting pentagon was replaced with an infinity diagram). Although not yet used for the differential diagnosis of DLB, sensitivity and specificity of the ACE‐III for identifying other dementias (AD and FTD) have shown favourable comparability with the ACE‐R.^34^

Our study has clinical implications. The ‘memory/visuospatial’ ratio was calculated from routinely collected ACE‐R data, therefore providing a simple cut‐off score that could be used by clinicians to assist diagnosis. Improving the diagnostic accuracy of DLB has several advantages. First, treatment strategy could be optimised, such that cholinesterase inhibitors may be introduced, whereas anticholinergics and neuroleptic medications should be carefully monitored or avoided.^1^, ^5^ Second, receiving an accurate diagnosis earlier is important. As individuals with DLB spend almost four additional days in hospital per year than individuals with AD,^35^ earlier diagnosis could minimise the number of acute admissions, thereby reducing inpatient costs and improving patient wellbeing. It may also help alleviate anxiety precipitated by the onset of neuropsychiatric, movement disorder or autonomic symptoms not seen in AD. Finally, increasing the true positive diagnostic rate would ensure selection of appropriate volunteers for participation in clinical trials of potential anti‐dementia therapies.

Overall, this study confirmed the reliability of the ACE‐R for detecting dementia in a pooled clinical sample, which further showed good discrimination between DLB and AD. These findings demonstrate that the proportion of impaired memory to visuospatial ability could be extended from the CAMCOG to the ACE‐R in the assessment of DLB. Markers of cognitive decline are key indicators of disease severity and progression that could also be used to calculate a simple cut‐off score to assist diagnosis. Future studies could combine cognitive performance with converging clinical and fluid biomarkers of DLB as well as structural and positron emission tomography (PET) imaging to test a predictive model built using multimodal features (e.g., by including visual hallucinations^31^; overlapping neuropsychiatric symptoms^36^; striatal dopamine transporter imaging^2^). Early and accurate clinical diagnosis of DLB helps optimise decision‐making and disease specific management in order to achieve the best possible outcome for those affected.

## CONFLICTS OF INTEREST

The authors declare no conflicts of interest related to this study.

## DATA AVAILABILITY

The data that support the findings of this study are available from the corresponding author upon reasonable request.
